# T3E: a tool for characterising the epigenetic profile of transposable elements using ChIP-seq data

**DOI:** 10.1186/s13100-022-00285-z

**Published:** 2022-11-30

**Authors:** Michelle Almeida da Paz, Leila Taher

**Affiliations:** Institute of Biomedical Informatics, Graz University of Technology, Graz, Austria

**Keywords:** Transposable element, ChIP-seq, Enrichment, Background signal, Input control, Epigenetics

## Abstract

**Background:**

Despite the advent of Chromatin Immunoprecipitation Sequencing (ChIP-seq) having revolutionised our understanding of the mammalian genome’s regulatory landscape, many challenges remain. In particular, because of their repetitive nature, the sequencing reads derived from transposable elements (TEs) pose a real bioinformatics challenge, to the point that standard analysis pipelines typically ignore reads whose genomic origin cannot be unambiguously ascertained.

**Results:**

We show that discarding ambiguously mapping reads may lead to a systematic underestimation of the number of reads associated with young TE families/subfamilies. We also provide evidence suggesting that the strategy of randomly permuting the location of the read mappings (or the TEs) that is often used to compute the background for enrichment calculations at TE families/subfamilies can result in both false positive and negative enrichments. To address these problems, we present the Transposable Element Enrichment Estimator (T3E), a tool that makes use of ChIP-seq data to characterise the epigenetic profile of associated TE families/subfamilies. T3E weights the number of read mappings assigned to the individual TE copies of a family/subfamily by the overall number of genomic loci to which the corresponding reads map, and this is done at the single nucleotide level. In addition, T3E computes ChIP-seq enrichment relative to a background estimated based on the distribution of the read mappings in the input control DNA.

We demonstrated the capabilities of T3E on 23 different ChIP-seq libraries. T3E identified enrichments that were consistent with previous studies. Furthermore, T3E detected context-specific enrichments that are likely to pinpoint unexplored TE families/subfamilies with individual TE copies that have been frequently exapted as *cis*-regulatory elements during the evolution of mammalian regulatory networks.

**Conclusions:**

T3E is a novel open-source computational tool (available for use at: https://github.com/michelleapaz/T3E) that overcomes some of the pitfalls associated with the analysis of ChIP-seq data arising from the repetitive mammalian genome and provides a framework to shed light on the epigenetics of entire TE families/subfamilies.

**Supplementary Information:**

The online version contains supplementary material available at 10.1186/s13100-022-00285-z.

## Background

Transposable elements (TEs) are repetitive sequences that comprise about half of the human genome and constitute large portions of other eukaryotic genomes [1]. TEs have been hierarchically classified into classes/subclasses depending on their mechanisms of transposition and chromosome integration, and into families/subfamilies according to sequence conservation and phylogenetic relationships [2]. Thus, several subfamilies are derived from the renowned Alu family, which belongs to a subclass of retroelements entitled SINEs (short interspersed nuclear elements) of class 1 (retrotransposons). Although a few subfamilies of TEs among the Alu and other families such as long interspersed nuclear element-1 (LINE-1) and SINE-VNTR-Alu (SVA) remain mobile in the human genome [3, 4], most TEs have lost their ability to transpose [5]. Nevertheless, TEs remain drivers of genome evolution, for example, as a source of novel *cis*-regulatory elements [6, 7]. Indeed, TEs contain transcription factor binding sites (TFBS) and many TE sequences have been co-opted as enhancers and alternative promoters [8, 9]. Additionally, TEs have been shown to modify the expression of nearby genes by acting as silencers or insulators [10, 11]. *Cis*-regulatory function in a particular biological context is typically assessed by chromatin immunoprecipitation followed by high-throughput sequencing (ChIP-seq) [12]. Thus, studies aiming to uncover how TEs rewire the regulatory network generally use ChIP-seq technologies to quantitatively analyse the enrichment of TEs on regulatory regions. However, the repetitive nature of TE-derived sequences poses a challenge for ChIP-seq-based studies [13].

Sequencing reads originating from TEs often map to multiple loci (“multimappers”) and cannot be unambiguously assigned to one locus. This is especially true for reads arising from the loci of “young” TE families/subfamilies, whose sequences have not had time to accumulate many mutations and are remarkably similar [14]. The problem has been tackled in different ways, for instance, by simply discarding multimappers from the analysis –the policy is adopted by many large consortia, including ENCODE [15]. This increases the specificity of the analysis, but discards a large proportion of the reads originating from TEs. Moreover, because of their sequence properties, discarding ambiguously mapped reads and using only uniquely mapping reads (“unimappers”) tends to affect the study of young TE families/subfamilies more than that of old ones [16, 17]. Another common and also simple strategy when dealing with TE-derived sequencing reads consists of randomly reporting one mapping [18]. This approach increases the number of mapped reads, but at the cost of precision [17]. Finally, more sophisticated TE-centric approaches have been developed, such as the one proposed by Chung et al. [19], which probabilistically reassign ambiguously mapped reads to regions where unimappers have been mapped, e.g., using the expectation-maximization (EM) algorithm. Nonetheless, this approach relies on the unambiguously mapped reads and seems to bias particularly towards regions that have any uniquely mapping read content [16]. Therefore, the adopted approach to deal with the mapping ambiguity issue impacts on quantifying the contribution of read mappings to a particular TE family/subfamily by counting the number of read mappings (or peaks) and enrichment analysis.

The most widely used tool for analysing TE families/subfamilies is RepEnrich [20], which addresses the ambiguously mapping matter using a strategy of fractional counts. RepEnrich first maps the reads to the reference genome in order to identify unimappers and multimappers. Then, the unimappers are checked for overlap with annotated TEs, whereas multimappers are remapped to pseudogenome assemblies for each TE family/subfamily containing the annotated individual copy sequences. The fractional count for each TE family/subfamily is computed as the sum of unimappers overlapping the given TE family/subfamily and the counting of multimappers weighted by 1/*n*, where *n* is the number of TE families/subfamilies a given multimapper mapped with. Hence, RepEnrich attempts to overcome the mapping ambiguity of multimappers by mapping them against individual copies and counting for TE families/subfamilies. Since some multimappers may have originated from non-annotated TE regions of the genome and map to those with a higher score [17], the procedure adopted by RepEnrich to map multimappers onto a pseudogenome containing only the individual copies of a TE family/subfamily (and other repetitive sequences) is likely to overestimate read counts for specific TE family/subfamily sequences. The available tools applied to the study of TEs differ in their strategy to distinguish reads originated from particular TE families/subfamilies. Instead of using a pseudogenome comprehending individual copy sequences for each TE family/subfamily such as in RepEnrich, Sun et al. [21] first map the ChIP-seq reads to the unmasked reference genome, extract reads that map to the annotated L1HS genomic sites and then exclude partiallycknowledgements mapped reads or reads with indels or with more than three mismatches. Subsequently, the filtered reads are remapped to the consensus sequence of the L1HS TE family to solve the issue of ambiguously mapping reads for the youngest human L1 family. This strategy seems beneficial for the youngest TE families/subfamilies counting estimation, albeit particular attention should be taken to interfamily ambiguities, in which reads derived from related TE families/subfamilies tend to map to the provided consensus sequence with suboptimal score compared to the entire genome, but still above the considered threshold score [14]. Contrary to young TE families/subfamilies, older TE family/subfamily sequences have accumulated more mutations and, thus, can diverge substantially from their consensus sequence.

Like other next-generation sequencing approaches, ChIP-seq experiments are subject to library preparation and sequencing biases. To identify and adjust for such biases, ChIP-seq datasets are analysed together with a control library obtained by sequencing the “input” control DNA of the corresponding experiment without ChIP. Besides correcting for background bias, when estimating the count of a particular TE family/subfamily, linear scaling of read depth is often performed to make the ChIP-seq sample library comparable with its control library. Thus, most approaches normalise the ChIP-seq sample against the measured background (input control DNA) [18, 21]. In the context of TE analysis, enrichment is often calculated against a background simulated by randomly permuting the locations of the read mappings (or of the TEs) in the genome [18, 21]. However, because the distribution of the input reads is not uniform, this strategy has been reported to produce artificial enrichments at TEs [22].

Because mapping ambiguities and inadequate TE enrichment analysis can produce misleading biological conclusions [17, 22, 23], new computational tools must be developed to overcome the pitfalls described above. Here, we introduce the Transposable Element Enrichment Estimator (T3E), an algorithm that identifies TE families/subfamilies featuring enrichment for specific targets of ChIP-seq data. In a sense, the strategy of T3E is similar to that of RepEnrich in that it relies on the reads mapping to individual TE copies to compute the number of read mappings associated with an entire TE family/subfamily. However, differently from RepEnrich, our algorithm maps ChIP-seq reads to the entire genome of interest without subsequently remapping the reads to particular consensus or pseudogenome sequences. Also, in its calculations T3E considers the number of both repetitive and non-repetitive genomic loci to which each multimapper mapped, rather than only the number of different TE copies to which it mapped. Additionally, in contrast to RepEnrich, we do not rely on a threshold for the number of base pairs a given read mapped on a TE family/subfamily, but rather, we account only for the portion of the read that mapped on the given TE family/subfamily. To adjust for library preparation and sequencing biases, our strategy shuffles positions according to the probability of a read starting at a given position on the input control DNA. Thus, to reflect the expected distribution of the read mappings in the input set, we assess the expectation from the read mapping distribution of the input control DNA. Moreover, we estimate the enrichment of TEs by comparing the read mapping counting in the ChIP-seq sample and in the average of the input library simulations for each studied TE family/subfamily, without any normalisation step. Finally, we applied our strategy to different ChIP-seq datasets to demonstrate the robustness of T3E and the reproducibility of the results obtained with our approach.

## Results

### Ambiguous mappings can lead to biases in the enrichment estimation at young TE families/subfamilies

**Fig. 1:**
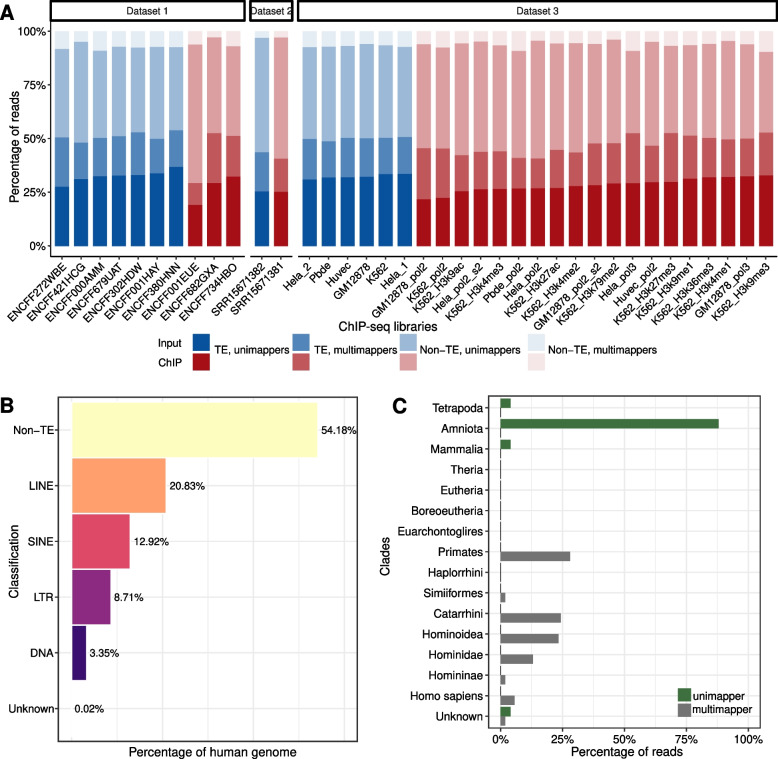
Young TE families/subfamilies are mainly covered by multimappers. **A** Fraction of reads mapping to TEs and regions not annotated as TEs (non-TE) in the human genome, separated into uni- and multimappers, for 37 ChIP-seq libraries (red) and their input controls (blue) (Datasets 1–3; Methods). File accession names are indicated on the x-axis. Individual copies belonging to the same TE family/subfamily are usually not identical. **B** Percentage covered by TE classes and non TEs in the human genome. TEs with unknown classification are reported as “Unknown”. The fraction of the human genome not covered by TEs is indicated by “non-TE”. Nearly half of the human genome comprises TEs. **C** Fraction of uni- (green) and multimappers (grey) mapping to TEs present in different eukaryotic clades. A TE family/subfamily was associated with unimappers (multimappers) if at least 90% of the reads mapping to its copies were unimappers (multimappers) in all ChIP-seq samples and input controls of Dataset 1. The vast majority of unimappers map to TEs that expanded in the common ancestor of amniotes (~ 310 million years ago); in contrast, most multimappers are associated with TEs that expanded in the earliest primates (~ 85 million years ago) and their descendants

To investigate the TE families/subfamilies associated with ambiguously mapping reads, we analysed the genomic distribution of the read mappings from 37 ChIP-seq experiments (Methods, Fig. 1A). Specifically, we considered 1159 different TE families/subfamilies covering 45.82% of the human genome (Fig. 1B) and 1133 distinct TE families/subfamilies covering 40.94% of the mouse genome (Supplementary Fig. S1). In general, libraries with longer read lengths had smaller proportions of multimappers, i.e., reads mapping to multiple genomic locations. Nevertheless, within the analysed range (26 bp–100 bp), we did not observe any substantial differences (Supplementary Fig. S2). We found that 16.34–32.41% of the reads were multimappers. Interestingly, 11.36–39.44% of multimappers mapped to regions of the genome that are not annotated as TEs. Hence, although most of the multimappers mapped to annotated TEs, a considerable fraction of them mapped to other repetitive sequences or non-annotated TEs. Additionally, 47.95–68.44% of reads mapping to TEs were unimappers, i.e., they only mapped once in the genome. This finding indicates that despite the repetitive nature of TEs, individual copies of the same TE family/subfamily are often not identical.

To examine the similarity among the TE families/subfamilies individual copies in more detail, we compared the TE families/subfamilies associated with multimappers and unimappers (Methods). Approximately 9% (107 out of 1159) of the TE families/subfamilies were predominantly covered by multimappers (i.e., more than 90% of the reads mapping to these families were multimappers). Remarkably, all these TE families/subfamilies were exclusive to primates and their derived clades (Fig. 1C), and 57% (61 out of 107) of them belonged to some of the youngest TE families/subfamilies, including LINE-1, SVA and many members of the Alu family. On the other hand, 2.5% (29 out of 1159) TE families/subfamilies were predominantly covered by unimappers (i.e., more than 90% of the reads mapping to these families were unimappers), and all of these TE families/subfamilies expanded in the vertebrate genome before the evolution of mammals (Fig. 1C). Notably, these TE families/subfamilies were present especially in Amniota (75.86%), with UCONs (ultra-conserved element) representing almost 70% of these TE families/subfamilies. These observations suggest that distinct strategies dealing with ambiguously mapped reads may lead to different conclusions for the functional analysis of TEs, depending on the age of the TE families/subfamilies. More specifically, using the strategy of discarding multimappers is likely to miss functions linked to young TE families/subfamilies.

### Uniform background distribution may lead to false positive and false negative enrichments at TEs

Next, we setup to study enrichment biases arising from the approach that calculates the background by randomly permuting the locations of the read mappings in the genome. Basically, this strategy assumes a uniform distribution of the read mappings.

**Fig. 2:**
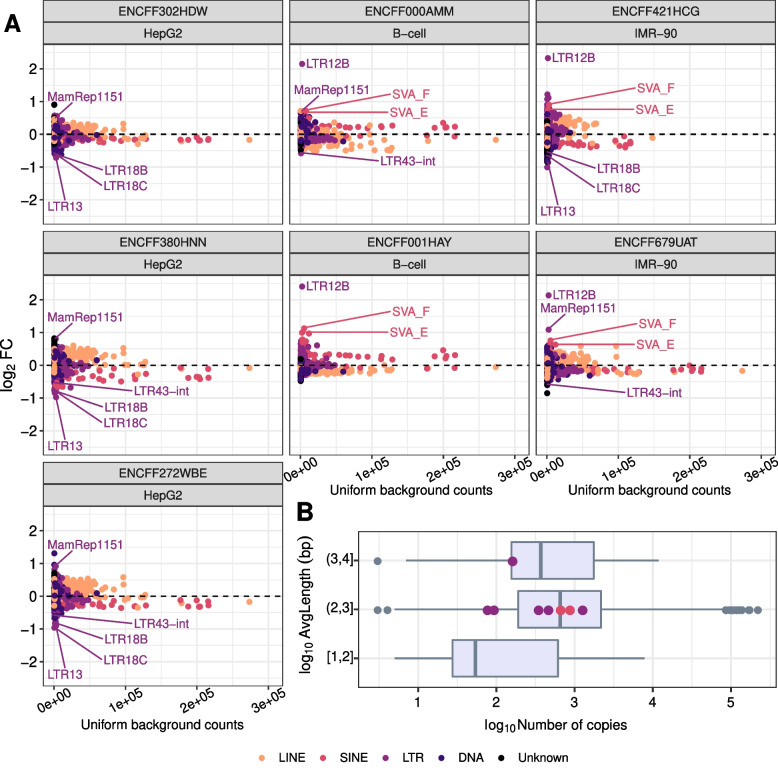
Uniform background distribution creates artifact enrichments for TEs. **A** Each dot represents a TE family/subfamily; TEs with unknown classification are reported as “Unknown”. Fold-Changes (FC) were calculated as the ratio between the average number of read mappings to a TE family/subfamily across 100 simulations that assume a uniform distribution of the mappings in the genome and the number of read mappings for the input control library. Dashed lines show a log_2_ FC = 0. Seven ChIP-seq input control experiments (Dataset 1) are presented. Outliers in four of seven input controls are highlighted. Several TE families/subfamilies presented deviations in the expected number of read mappings from the input control library using uniform background distribution approach. **B** Numbers of individual copies and their average lengths for distinct TE families/subfamilies in the human genome. Four underestimated and four overestimated TE families/subfamilies are highlighted with the colours of their classification in the boxplot

Consistent with our expectations, we found that although the relationship between the number of read mappings simulated assuming a uniform distribution and the number of read mappings observed for the input control was linear (Fig. 2A), there were clear deviations for specific TE families/subfamilies. Notably, compared to the input control, assuming a uniform distribution led to 30% less read mappings for LTR13, LTR18B, LTR18C and LTR43-int subfamilies, and to 50% more read mappings for LTR12B, MamRep1151, SVA E and SVA F subfamilies in at least four of the seven ENCODE ChIP-seq input controls (Dataset 1; see Methods and Supplementary Table S1). In general, we observed that the TE families/subfamilies for which the number of read mappings was underestimated by assuming a uniform distribution comprise shorter TEs than those of families/subfamilies for which the number of read mappings was overestimated; also, the former are smaller (i.e., have fewer individual copies in the genome) than the latter. Notwithstanding, the TE families/subfamilies that are underestimated/overestimated do not differ in any obvious way from others in terms of their size and length of their individual TE copies (Fig. 2B). Thus, while the TEs in the underestimated long-terminal repeat (LTR) subfamilies vary from 301 to 871 bp per copy, TEs in the overestimated LTR12B subfamily are, on average, 1967 bp long. Additionally, while there are only 76 and 92 copies of TEs of the LTR18C and LTR43-int subfamilies, respectively, the LTR subfamily MamRep1151 has with 1251 copies more than twice the median number of copies of all TE families/subfamilies in the human genome. Therefore, using the uniform distribution as background for enrichment analysis may result in both false positive and negative enrichments that cannot be easily predicted.

### T3E is a novel approach to estimate enrichment at TEs from ChIP-seq data

**Fig. 3:**
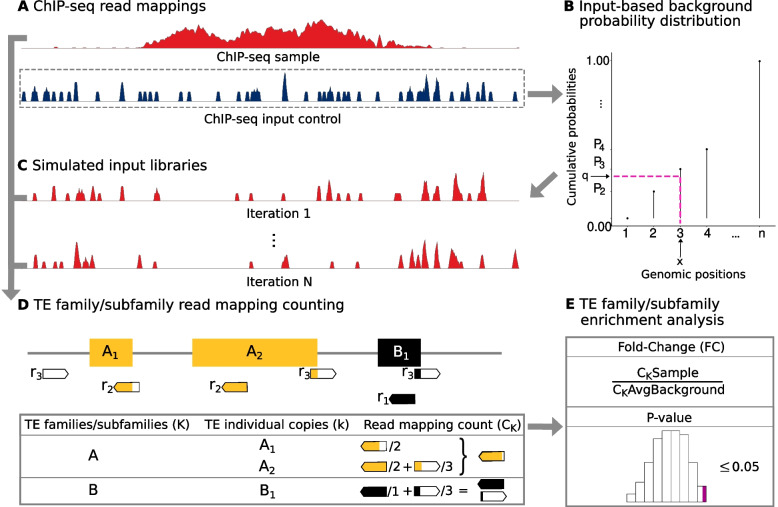
Overview of the T3E workflow for TE families/subfamilies enrichment estimation. **A** Read mappings from ChIP-seq and input control libraries are used as input for T3E. Note that mapping must be performed with a tool able to report all read mappings, including those that are ambiguous. **B** T3E estimates a probability for each position in the genome from the distribution of read mappings for the input control library. **C** T3E samples reads according to the probabilities estimated in (B) to generate *N* input libraries that are used to compute a *P*-value and a Fold-Change (FC). Every simulated input library has the same size as the ChIP-seq sample. **D** Read mappings are counted for ChIP-seq sample and every simulated input library. The read mapping count associated with the *K*th TE family/subfamily (*C*_*K*_) is given by the sum of the portions (percentages of the length) of the reads that map to each of its TE copies, each weighted by the ambiguity of the corresponding mapping. **E** The FC of the *K*th TE family/subfamily is calculated as the ratio between the number of read mappings for the sample (C_K_Sample) and the average number of read mappings across all *N* simulated input libraries (C_K_AvgBackground)

To address the issues presented above, we developed T3E (Methods). Acknowledging technical limitations when it comes to characterising specific TE instances in the human genome, T3E computes enrichments for entire TE families/subfamilies, rather than for their individual TE copies. The algorithm uses the genomic distribution of the read mappings of the ChIP-seq input control experiment (Fig. 3A) to estimate a background probability distribution (“input-based background probability distribution”, Fig. 3B). Specifically, the probability of observing a mapping at a given genomic position is assumed to reflect the coverage of the input control. T3E then samples from this probability distribution to construct an appropriate background for enrichment calculations (“simulated input library”, Fig. 3C). Consequently, the expected number of read mappings that T3E estimates for each TE family/subfamily is proportional to that observed in the input control (Supplementary Fig. S3). To account for the ambiguity of read mappings, T3E *weights* each read mapping to a TE copy by the number of genomic loci to which the read maps, so that the contribution of a particular read mapping to the total number of read mappings associated with that TE copy is inversely proportional to the overall number of loci to which the corresponding read maps (Fig. 3D). Moreover, the calculation is done at the single nucleotide level, i.e., only the number of nucleotides from a read mapping to a TE copy are considered. The process is repeated multiple times in order to obtain an empirical *P*-value. Finally, a Fold-Change is calculated as the ratio between the number of read mappings associated with the TE family/subfamily of interest in the ChIP-seq sample library and the average number of read mappings associated with the same TE family/subfamily across all simulated input libraries (Fig. 3E). T3E is a novel, open-source framework for the epigenetic analysis of TE families/subfamilies from which the entire TE community could benefit.

The run-time and memory complexities of T3E depend on the library size, number of ambiguously mapping reads, and number of genomic loci to which ambiguously mapping reads map. In the worst-case scenario, the library would contain only multimappers, each mapping to every single genomic locus. In this case, the theoretical complexity is proportional to *genome size* × *input library size* × *sample library size*. However, on an average ENCODE ChIP-seq sample (HepG2, see Methods), the algorithm showed approximately linear run-time and memory complexities with respect to the input library size (Supplementary Fig. S4).

### T3E detects biologically-relevant TE family/subfamily enrichments

To test how T3E can be used to characterise the epigenetic profile of TE families/subfamilies we analysed three ChIP-seq libraries and their seven input control libraries generated by the ENCODE consortium (Dataset 1). The experiments involved ChIP-seq against H3K4me3 –a histone mark associated with euchromatin– in B-cells –responsible for humoral responses of the immune system–, H3K79me1 –also associated with euchromatin– in a cell line isolated from normal lung fibroblast tissue (IMR-90), and the transcription factor FOXP1 in a cell line isolated from hepatocellular carcinoma (HepG2).

Few TE families/subfamilies show enrichment for all three types of samples, perhaps indicating some sort of basic level of enrichment. Specifically, we found that the LTR12E (FCs between 1.14 and 9.19), LTR13 (FCs between 1.32 and 2.01) and MER57E3 (FCs between 1.26 and 7.58) subfamilies of the long terminal repeats (LTRs) are enriched in all three cell types, even though at different levels. This observation is consistent with reports about the LTRs of endogenous retroviruses (ERVs) frequently having been exapted as gene promoters [24], which are often not cell-specific.

**Fig. 4:**
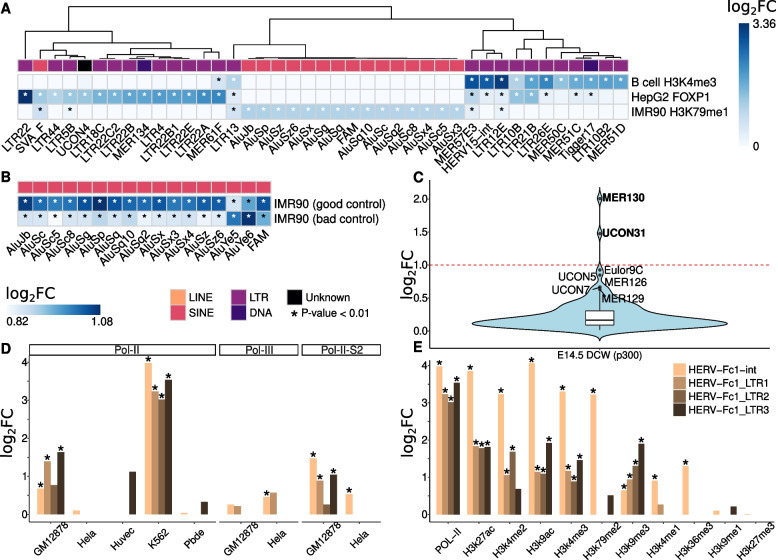
T3E characterises epigenetic profile of TE families/subfamilies in cell type-specific scenarios. **A** The heatmap shows the FCs of the TE families/subfamilies that T3E detected as enriched (*P*-value < 0.01) with FC > 2 in at least one ChIP-seq experiment of Dataset 1. *P*-values < 0.01 are indicated by asterisks; FCs > 2 are shown with white asterisks. White-coloured cells represent no signal. Columns are clustered using Euclidean distance and complete linkage. TE families/subfamilies are enriched in a cell type- and ChIP target-specific manner. Mainly SINEs are enriched in the IMR-90 cell line and essentially LTRs are enriched in B-cell and HepG2 cell types. **B** The heatmap shows the FCs of the TE families/subfamilies that T3E detected as enriched (*P*-values < 0.01) with FC > 2 in at least one of two different ChIP-seq input controls. White asterisks indicate FC > 2. Input control quality impacts on the Fold-Change level of enrichments but does not change the biological interpretation of the results. **C** Enrichment for the transcriptional co-activator p300 in the dorsal cerebral wall (DCW) of E14.5 mouse embryos. Only 130 (out of 997) TE families/subfamilies with *P-*value < 0.01 are represented. Mild outliers (lying between 1.5 times and 3 times the interquartile range above the third quartile) are indicated by their names; only MER130 and UCON31 feature FC > 2. Ancient TE families/subfamilies might have been co-opted as *cis*-regulatory elements important to the development of the mammalian neocortex. **D-E** Enrichment HERV-Fc1 subfamilies for polymerases and histone marks in two normal (Huvec, Pbde) and three transformed and cancer cell lines (Hela, GM12878, K562). Asterisks represent *P-*values < 0.01. HERV-Fc1 subfamilies (coloured differently) feature higher enrichment for Pol-II in the K562 CML cell line compared to other cell lines and active chromatin histone marks (H3K27ac, H3K4me2, H3K9ac, H3K4me3, H3K79me2, H3K4me1 and H3K36me3) present higher levels of HERV-Fc1 subfamilies compared with histone marks associated with repressed chromatin (H3K9me1, H3K9me3 and H3K27me3) for Pol-II in the K562

Supporting a functional association, we observed specific enrichment profiles depending on the cell type and ChIP target (Fig. 4A). For instance, six subfamilies of the same TE family, the endogenous retrovirus-K (ERVK), featured enrichment for FOXP1 in HepG2 cells, with Fold-Changes varying from 2.98 (LTR22C2) to 10.24 (LTR22) (Methods). These six subfamilies did not present enrichment in any of the other ChIP-seq samples. Similarly, most Alu retrotransposon subfamilies were only enriched for H3K79me1 in IMR-90 cells (FCs between 1.24 and 2.12). Moreover, the LTR21B and LTR10B subfamilies (FCs between 2.05 and 3.59) only showed enrichment in two out of three different cell types. Whereas the LTR21B subfamily, enriched both for H3K4me3 in B-cells (FC = 3.59) and for FOXP1 in the HepG2 cancer cell line (FC = 2.70) but not for H3K79me1 in the IMR-90 normal cell line (FC < 1, *P*-value = 0.85), has been associated with immune checkpoint activity and CD8^+^ T-cell expression in tumours [25], the LTR10B subfamily, also enriched for H3K4me3 in B-cells (FC = 2.05) and FOXP1 in HepG2 (FC = 2.53) cells but not in IMR-90 cells (FC = 1.01, *P-*value = 0.45), has been associated with tumour suppressor p53 protein binding along with the MER61 family [26]. Curiously, the MER61F subfamily was enriched for FOXP1 in HepG2 (FC = 4.68) and moderately enriched for H3K4me3 in B-cells (FC = 1.47) too. These enrichments indicate that TEs may act as *cis*-regulatory elements, contributing to gene regulation by functioning as enhancers, promoters, silencers or insulators.

The fact that the enrichments observed are cell-type specific and restricted to certain transcription factors and histone marks depending on the TE family/subfamily strongly suggests that T3E is able to capture functional properties of the TEs and, hence, provides a useful framework for the systematic investigation of TEs’ *cis*-regulatory potential in a context-specific manner.

### TE enrichment estimations by T3E are robust to variation in the quality of the ChIP-seq input control

To evaluate the impact of the quality of the input control on the enrichment of TE families/subfamilies estimated by T3E, we applied T3E to the analysis of the libraries of a H3K79me1 ChIP-seq experiment performed by the ENCODE Project in IMR-90 cells its two input controls (Methods). One of the input control libraries had been audited by ENCODE in the “insufficient read depth” category (10,800,072 processed reads, file accession: ENCFF421HCG); the other has sufficient read depth (47,554,621 processed reads, file accession: ENCFF679UAT). For both input controls, Alu families featured the highest enrichments, with AluJ and AluS subfamilies featuring higher Fold-Changes for the input control with sufficient read depth (“good control”) and AluY subfamilies presented higher Fold-Changes for the input control with insufficient read depth (“bad control”). Specifically, we found that among the 1052 TE families/subfamilies with 50 or more individual TE copies in the human genome, 16 Alu retrotransposon subfamilies and one fossil Alu monomer (FAM) were considered enriched (*P*-value < 0.01) for both the good and the bad controls and had a FC of 2 or higher in at least one of them; in 15 cases, the Fold-Changes were higher for the good control (FCs between 2.01 and 2.12 for the good control vs FCs between 1.76 to 1.93 for the bad control), and in two cases the Fold-Changes were higher for the bad control than the good control (FCs between 2.02 and 2.11, Fig. 4B). Besides Alu retrotransposons, a few other TE families/subfamilies showed moderate enrichments (e.g., SVA retrotransposon subfamilies A-F, FLAM and FRAM, with FCs between 1.59 and 1.85), but their enrichment was always lower than 2-fold, independently of the read depth of the input control. Hence, within reasonable limits, the quality of the input control ChIP-seq data used by T3E to construct the background probability distribution is not likely to impact the biological interpretation of the enrichment analysis, but rather the Fold-Change thresholds chosen to define a TE family/subfamily as enriched.

### Mouse neocortex enhancers present modest enrichment for ancient repeats

Next, we applied T3E to ChIP-seq data of the dorsal cerebral wall (DCW) for the transcriptional co-activator p300 from mouse embryos (Dataset 2; see Methods and Supplementary Table S1), for which Wenger, Notwell and their colleagues [27, 28] had reported a striking enrichment (FC = 73) for the MER130 subfamily. The authors used a non-TE aware approach. In summary, they mapped the ChIP-seq reads to the mouse genome using ELAND [29] and retained only reads mapping uniquely with two or fewer mismatches. Peaks were called with MACS [30] based on uniquely mapping ChIP-seq reads and using input DNA reads as control. The expected enrichment for each repeat family/subfamily was calculated by randomly shuffling the locations of the resulting peak set 10,000 times across the entire genome and calculating the average. Only TE families/subfamilies with at least 50 individual copies were considered for this analysis, leading to 997 TE families/subfamilies. Consistent with the published observations, we found that MER130 was the TE subfamily exhibiting greatest enrichment, albeit with a much lower Fold-Change (FC = 4.03; Fig. 4C). Similarly, T3E detected enrichment for the UCON31 subfamily (FC = 2.79), for which the aforementioned studies had also indicated a much higher enrichment (FC ≈ 35). Despite the general agreement between our findings, we noticed some interesting differences. For example, two other TE families/subfamilies reported by Wenger et al. as highly enriched, MER124 (FC ≈ 15) and AmnSINE1 (FC ≈ 5) had only moderate Fold-Changes according to our analysis (MER124 with FC = 1.34 and AmnSINE1 with FC = 1.33). Further, we saw high Fold-Changes for six subfamilies, namely, Eulor9C, MER125, MER126, MER129, UCON5 and UCON7 (FCs between 1.51 to 1.91; Fig. 4C), which had not been acknowledged before. Eulors (euteleostomi conserved low frequency repeats), MERs (medium reiterated frequency repeats) and UCONs are ancient transposable elements [31]. Eulor families present self-complementary regions which suggest they might have been accumulated in the genome by DNA transposition just as some MER families [32]. Since most DNA transposable elements are predicted to be ancient immobilized elements in the genome [33] that have been transposed in an imprecise manner in the vicinity of genes, they might have been co-opted as transcriptional regulators of genes involved in developmental processes [34]. Similarly, UCON families are often located nearby or overlapping genes involved in regulation of transcription and development [35]. This suggests that a broader range of ancient TE families/subfamilies may have been exapted as enhancers and other *cis*-regulatory elements of genes involved in the development and evolution of the mammalian neocortex.

### RNA polymerases bind differently to LTR retrotransposons depending on the cell type

Finally, we applied T3E to an RNA polymerase II and III (RNA Pol II and Pol III) ChIP-seq dataset that had already been analysed with RepEnrich [20]. In agreement with the results obtained by Criscione et al. [20], T3E detected more TE families/subfamilies enriched for RNA Pol II in transformed cell lines (FCs between 2.05 and 17.89) than in normal cell lines (FCs between 2.03 and 3.37). In particular, we found the lowest number of enriched TE families/subfamilies (7 out of 1159) in the PBDE (peripheral blood-derived erythroblast) normal cell line and the highest number (62 out of 1159) in the K562 chronic myelogenous leukemia (CML) cell line. Criscione et al. observed that many LTR retrotransposons and some SINEs featured enrichment for Pol II (FCs > 2.83 and false discovery rates (FDR) < 0.05); on the contrary, only a few TE families/subfamilies of DNA and LINE retrotransposons did. This is especially true for transformed cell lines. Overall, Criscione et identified more than 100 retrotransposon TE families/subfamilies displaying enrichment for Pol II in transformed cells (GM12878 lymphoblastoid, Hela adenocarcinoma and K562) and at least 20 retrotransposon TE families/subfamilies in normal cells (human umbilical vein endothelial cell (HUVEC) and PBDE). Using T3E, we only observed a total of 24 TE families/subfamilies featuring enrichment for Pol II (FCs between 2.83 and 17.89) in transformed (22 out of 24) and normal cell lines (2 out of 24), whereby approximately 80% were LTR retrotransposons. Additionally, we did not find any LINE retrotransposon family/subfamily enriched for Pol II. Criscione et al. suggested that RNA polymerases bind to TE families/subfamilies in a cell line-specific manner. Indeed, T3E detected 13 TE families/subfamilies that were enriched in only one cell line. Nevertheless, we also observed TE families/subfamilies enriched in multiple cell lines, but with lower Fold-Changes in normal cell lines than cancer cell lines, suggesting systematic changes in their epigenetic profiles in cancer. For instance, we found that LTR12E was enriched for Pol II in both transformed and normal cell lines, although transformed cells tended to feature higher levels of enrichment (FCs between 4.55 and 9.57) compared to normal cell lines (FCs 2.07 and 2.19 for PBDE and HUVEC, respectively; Supplementary Fig. S5). This is consistent with the ability of some LTR12 subfamilies to recruit RNA polymerase II and act as cell-specific promoters in certain cell types in mammalian genomes [24, 36].

Criscione et al. highlighted that the human endogenous retrovirus HERV-Fc1 and its subfamilies were enriched for RNA Pol II and Pol II phosphorylated on serine 2 (Pol II S2). In particular, they noted that the internal region of HERV-Fc1 (int) was highly enriched for Pol II and Pol II S2 in the K562 CML cell line. Comparing the results of T3E with those of RepEnrich, we observed similar trends for HERV-Fc1, but differences for individual subfamilies. To illustrate, RepEnrich found enrichment for Pol II S2 at HERV-Fc1-int, LTR1 and LTR3 in the Hela cell line (FC > 2), while T3E detected moderate enrichment at HERV-Fc1-int (FC = 1.45) and no enrichment at any of the other HERV-Fc1 LTRs (Fig. 4D). Like RepEnrich, T3E identified enrichment for Pol II at HERV-Fc1-int in the K562 cell line (FC = 15.77); albeit, RepEnrich reported lower levels of enrichment (FC = 7). Additionally, the results of RepEnrich suggest that HERV-Fc1 is –with some exceptions– associated with histone modifications for active chromatin. The results obtained with T3E are similar, including the enrichment at HERV-Fc1 for the repressive mark H3K9me3 that was reported by the developers of RepEnrich (Fig. 4E), and the enrichment for Pol II and Pol II S2 at HERV-Fc1 in transformed and cancer cell lines (K562 and GM12878), which calls attention to the potential expression of HERVs in cancerous tissues [37]. Nonetheless, it is worth remarking that those enrichments are only supported by a few annotated individual TE copies (3 for LTR2, 5 for LTR3, 7 for the internal region and 17 for LTR1)**.** In conclusion, our data add to the growing body of evidence supporting the implication of TEs in important cellular processes.

## Discussion

A collaborative effort has been made in the last decade to advance our knowledge and understanding of the role of transposable elements as *cis*-regulatory elements and their contribution to development and disease. Although experimental technologies have improved and numerous computational strategies have been proposed, several challenges remain when studying the epigenetic profile of TEs, particularly when mapping TE-derived sequencing reads to the genome and analysing enrichment at TEs. Most of the computational tools developed to tackle TE-derived reads have been designed for RNA-seq data and aim to reveal patterns of TE transcription. Instead, ChIP-seq datasets enable the exploration of the epigenetic signatures of TEs. RNA-seq and ChIP-seq experiments are fundamentally different, and only a few tools are available that can properly handle ChIP-seq data. These tools mainly differ from each other in their strategies to account for ambiguously mapping reads and their targeted genomic feature (TE families/subfamilies, particular TE classes, or specific TE copies) [14]. Given that none of the proposed approaches constitute a perfect solution for the challenges faced when studying repetitive sequences [16], it is up to the researchers to decide which strategy is best suited for their particular question and experimental design.

The algorithm presented here, T3E, estimates the enrichment from ChIP-seq data at the level of TE families/subfamilies and not their individual copies. This is a pragmatic approach that acknowledges that the genomic origin of multimappers cannot be completely determined without ambiguity. In principle, using longer reads should reduce the number of multimappers. However, within the read length range analysed here, the observed differences were generally small to negligible (Supplementary Fig. S2). Indeed, we believe that this issue will only be effectively addressed with the advent of long-read sequencing technologies for genome-wide mapping protein-DNA interactions. Nevertheless, to lessen the impact of ambiguously mapping reads in enrichment calculations, T3E weights each read mapping by the overall number of loci to which the corresponding read maps in the genome. Next, to estimate the enrichment at TE families/subfamilies, T3E simulates a background distribution of read mappings by randomly sampling read mappings based on the structure of the input control library. The input control varies depending on the cell type and condition since chromatin accessibility also differs. Additionally, biases in the chromatin fragmentation and amplification procedures can lead to the over- or under-representation of certain sequences. As a result, the distribution of reads from a sequencing experiment across the genome is not uniform [38]. Our findings show that assuming a uniform background distribution has an impact on the expected number of read mappings associated with specific TE families/subfamilies. Consequently, the commonly used approach of randomly permuting the locations of the read mappings (or of the TEs) in the genome can be expected to lead systematic false positives and negatives. In particular, TE families/subfamilies of the LTR subclass are the most affected ones. By estimating the background distribution using the input control library, T3E accounts for potential biases arising during library preparation, avoiding systematic biases in the estimation of enrichment at TE families/subfamilies observed when the background distribution is constructed by assuming that the read mappings (or the TEs) are uniformly distributed across the genome. Another advantage of T3E is that it was designed in such a way that there is no need for scaling the read depth of the libraries for the computation of the enrichment, since the library sizes of the simulated background libraries and of the ChIP-seq sample experiment are the same. Thus, our approach does not suffer from artifacts inherent to the estimation of scaling factors [39], which may impact the biological interpretation of the results.

Naturally, T3E enrichment estimates depend on the quality of the data, in particular the quality of the input control library which is used to estimate the background distribution of read mappings. Nevertheless, we showed that even with a drastic reduction in read depth, the biological interpretation of the results is sustained. Further, assuming an average ChIP-seq library, T3E has an approximately linear time complexity with respect to the size of the input control library. When using large libraries, it is advisable to run T3E in a workstation with enough computational resources to handle the potentially much larger numbers of read mappings. It is the number of mappings –more generally, the structure of the library– what has the largest impact on the computational power required to run T3E. The input control library of Supplementary Fig. S4 has approximately 70 M reads, but those reads map to 903,809,170 locations in the human genome. If the number of mappings is high, we recommend down-sampling the input control, since we have shown that, within reasonable bounds (20 million reads), this does not lead to any substantial loss of information. Ultimately, the runtime will depend on the chosen number of iterations. Although we recommend 100 iterations, 10 iterations also produce good Fold-Change estimates. Generally speaking, we recommend that requirements and standards for ChIP-seq experiments suggested by the ENCODE consortium [15] are satisfied. If unusual extremely high coverages in heterochromatin genomic regions are found, most likely resulting from problems during chromatin fragmentation, using the optional T3E functionality to filter out regions of extremely high signals does not impact on the final TE families/subfamilies enrichments (Supplementary Fig. S6). In our analysis, we applied this functionality only for analyses of the mouse genome, which presents large portions of tandemly repeated sequences [40]. It was motivated by the presence of major satellites and simple repeats mainly in centromeric and telomeric regions which are systematically mapped with artifact read mappings. T3E uses BWA mem [41], but in principle, T3E could use a different mapping algorithm as long as it can be configured to report secondary alignments. Several mappers support this and offer an option to ensure that a relatively large number –if not all– of all possible mappings of a read are reported. Such mappers include Bowtie2 [42] (“-a” mode) Novoalign [43] (“-r All”), and BBMAP [44] (“secondary = t”). In principle, they all could be used with T3E. Moreover, mappers typically rely on a large number of additional parameters. Here, we simply opted for using BWA mem with default parameters. Optimally setting other parameters would require considering properties of the ChIP-seq experiment such as read length, read quality, and the quality of the available reference genome. For instance, using BWA mem but increasing the minimum seed length (the default is “-k 19”) reduces the number of secondary mappings at the cost of decreased mapping sensitivity and overall number of read mappings (Supplementary Fig. S7). Thus, T3E makes it possible to study the epigenetic profile of TE families/subfamilies using ChIP-seq data.

By applying T3E to ChIP-seq libraries of a variety of cell types and epigenetic marks, we hereby demonstrated the reproducibility and robustness of the results obtained with our approach. By re-analysing datasets that had been previously examined by other studies, we showed that the findings of T3E are generally consistent with those published by other authors. Although we did not necessarily detect enrichment for exactly the same TE families/subfamilies (or at the same level), we indeed noted similar trends which led to the same biological conclusions. Our observations were also in agreement with those obtained by non-TE aware strategies, although large differences were evident in the magnitude of the Fold-Changes – which appears reasonable, since they do not have the same interpretation. Finally, T3E identified additional TE families/subfamilies which are potentially involved in central cellular and developmental processes. Our findings corroborate that many TE families/subfamilies have contributed to the evolution of mammalian regulatory networks and play important cell-type-specific *cis*-regulatory roles.

## Conclusions

Here we introduced T3E, an open-source framework for the study of regulatory roles of TEs at a level of families/subfamilies. T3E proposes a solution to challenges concerning the assignment of the ambiguously mapping reads that are typically derived from TEs and the estimation of a background for enrichment analysis. We applied T3E to confirm results from previous studies, but also to point out differences, such as consistent enrichment patterns for the Eulor9C, MER125, MER126, MER129, UCON5 and UCON7 families/subfamilies, whose epigenetic roles remain unexplored.

## Methods

### Publicly available ChIP-seq datasets

We considered three different single-ended sequencing ChIP-seq datasets which are detailed in Supplementary Tables S1 and S2. We downloaded the raw data (FASTQ files) using UCSC Genome Browser Download Server [45], ENCODE Project data repository [46] and Gene Expression Omnibus data repository [47].

### Repeat annotation

Repeat annotation for the human (GRCh38/hg38) and mouse (GRCm38/mm10) genome assemblies were retrieved from the RepeatMasker track of the UCSC Genome Browser [45, 48]. Repeat annotations were processed to filter out simple repeats (micro-satellites), satellite DNA, low complexity sequences, RNA repeats (including RNA, tRNA, rRNA, snRNA, scRNA, srpRNA) and non-TE elements. TE families/subfamilies were defined based on the “repFamily” column of the RepeatMasker track of the UCSC Genome Browser, which is based on Wicker et al’s classification [2]. In this classification, a family is defined as a group of TEs that share 80% (or more) sequence identity in at least 80% of their coding region, internal domain, or within their terminal repeat regions. The subfamilies are subpopulations of large families that can be clearly segregated. Alu is an example of a TE family; AluJb and AluSc are subfamilies of the Alu family. Our study comprehends a total of 35 and 32 families in the human and mouse genomes, respectively, and 1159 and 1133 subfamilies in the human and mouse genomes, respectively. Overlapping and immediately adjacent TE individual copies of the same TE family/subfamily were merged to produce the final annotation.

### Quality control and mapping of ChIP-seq reads

The quality of all raw ChIP-seq samples and their respective input controls (FASTQ files) was assessed using FASTQC v0.11.9 [49]. Sequences were trimmed or filtered out for sequencing adapters, low-quality reads (minimum Phred score of 15) and IUPAC nucleotide “N” (representing any base) using BBduk (version from November, 2019) [50] and Trim Galore v0.6.4_dev [51]. Reads were mapped to the human (GRCh38/hg38) or mouse (GRCm38/mm10) reference genome assemblies using BWA mem v0.7.17 [41] with the parameter “-a”, which outputs all found mappings. All other parameters were set to their default values, except for the score “-T” which was reduced to 25 for samples with read length below 30 bp. The read length was calculated as the average of 10,000 reads of the library. Genome sequences in FASTA format were downloaded from UCSC Genome Browser [45, 48]. Duplicate reads were removed using PICARD v2.24.0 [52]. Remaining read mappings were processed using SAMtools v1.10 [53] and BEDtools v2.27.1 [54] to filter out reads mapping to the mitochondrial chromosome, unmapped reads, and non-chromosomal scaffolds. For Dataset 3, BAM files for replicates of the same condition were pooled together using SAMtools merge. Large sequencing libraries were randomly down sampled to 20 million reads to reduce the dataset to a more manageable size for T3E computational calculations.

### Mapping loci assessment

The genomic locations where reads mapped on were assessed and, in order to investigate TE families/subfamilies most mapped by reads ambiguously or unambiguously, we considered the TE families/subfamilies in which more than 90% of the mapped reads were multimappers or unimappers, respectively. For this analysis, we considered TE families/subfamilies following the selection criteria among all samples and input controls of ENCODE ChIP-seq experiments (Dataset 1). To identify the clades and species in which the selected TE families/subfamilies have been found, we used Dfam web browser [55].

### Filtering artifactual genomic regions on the mouse genome

Dataset 2 mapped on the mouse genome featured major satellite and single repeat regions with unusual extremely high coverage, potentially from chromatin accessibility bias in heterochromatic genomic regions. In order to systematically exclude such regions, we calculated the number of read mappings in sliding windows of size 100 and step 50. Then, we identified the windows with read mappings above the 99.997th percentile for both chromosome and genome (Supplementary Fig. S8), and excluded the corresponding genomic regions from further analyses. Windows with zero counts were not considered for the calculation.

### Input-based background probability distribution

Our method estimates the probability of a read starting at an effective genomic position *n* on a given chromosome *c* as a fraction of the coverage of read mappings in the given position *n* and the coverage of read mappings in the entire chromosome *c*. Let *S* designate the set of all reads which map to chromosome *c*, |*S*| be the number of reads in *S* and *r*_1_, *r*_2_, …, *r*_|*S*|_ be the reads in *S*. We estimate the coverage of read mappings in the position *n* (as)

$${\mathit{\operatorname{cov}}}_n=\sum_{i=1}^{\left|S\right|}\frac{I_n\left({r}_i\right)}{N_{r_i}}$$

where

$${I}_n\left({r}_i\right)\left\{\begin{array}{c}1,\textrm{if}\ {r}_i\ \textrm{maps}\ \textrm{on}\ n\ \\ {}0,\kern0.5em \textrm{otherwise}\end{array}\right.$$

and ${N}_{r_i}$ is the number of distinct genomic locations to which *r*_*i*_ maps. Let *C* denote the set of effective positions on the chromosome *c*, |*C*| be the number of effective positions in *C* and *n*_1_, *n*_2_, …, *n*_|*C*|_ be the effective positions in *C*. We estimate the coverage of read mappings in the chromosome *c* (as)

$${\mathit{\operatorname{cov}}}_c=\sum_{n\in C}{\mathit{\operatorname{cov}}}_n$$

Thus, the coverage is inversely proportional to the number of read mappings observed for the input control library mapping. More precisely, the probability of a read starting at *n* is defined as:

$${p}_n=\frac{{\mathit{\operatorname{cov}}}_n}{{\mathit{\operatorname{cov}}}_c}$$

Nucleotide positions to which no reads were mapped had probability of zero. The probability associated with each nucleotide of the effective genome was used to create the simulations for estimating the expected read mapping counting for each TE family/subfamily.

### Simulated input libraries

An arbitrary genomic position was randomly selected according to the input-based background probability distribution described above. More precisely, this position was chosen from the effective genome, defined as the genomic regions for which input control coverage is greater than zero. Let the random genomic position be represented by the discrete random variable *X* (random variate *x*) where *p*_*n*_ = *p*(*X* = *n*) and let the cumulative probability be *P*_*n*_ = *p*(*X* ≤ *n*). Let *q* be a random number, if *P*_*n* − 1_ < *q* < *P*_*n*_ then *x* = *n*. Next, among the reads of the input control library mapping at the random genomic position *n*, we randomly selected one using the NumPy (v1.19.4) random-choice function using as parameter the probabilities associated with each read. Let *r*_*i*_ be the *i*th read which maps on the selected position *n*, we estimate the probability of a given read *r*_*i*_ (as)

$${p}_{r_i}={\left({\mathit{\operatorname{cov}}}_n{N}_{r_i}\right)}^{-1}$$

Then, the selected position *n* is shifted to be in the center of the selected read *r*_*i*_. The difference in base pairs of the shift is used to adjust the starting positions of each of the read mappings of *r*_*i*_. In such a manner, we obtain a symmetrical simulation of the input control, adding a systematic variation to the simulated input library. This procedure is performed as many times as there are reads in the ChIP-seq sample library of interest, resulting in a simulated input library of the same size as the ChIP-seq sample.

### TE family/subfamily read mapping counting and enrichment analysis

The contribution of a read mapping to the counting of TE family/subfamily was assumed to be inversely proportional to the number of mappings for the same read in the entire genome, taking into account the portion of the read that actually mapped to each individual copy of the TE family/subfamily under consideration, as follow:

$${C}_K=\sum_{k\in K}\sum_{r\in S}\sum_{i=1}^{N_{r_i}}\frac{l_{k\ {r}_i}}{N_{r_i}\ {L}_{r_i}}$$

where *K* is the set of all individual copies of a TE family/subfamily in the genome, *S* is the set of all reads in the ChIP-seq library, ${N}_{r_i}$ is the number of mappings of read *r*_*i*_, ${L}_{r_i}$ is the length of read *r*_*i*_, and ${l}_{k\ {r}_i}$ is the number of nucleotides of the *i* th mapping of read *r*_*i*_ overlapping with TE individual copy *k*, where $\left\{{l}_{k\ {r}_i}\in {\mathbb{Z}}_0^{+}:0\le {l}_{k\ {r}_i}\le {L}_{r_i}\right\}$. Enrichment was calculated for TE families/subfamilies, not for individual TE individual copies of the families/subfamilies. For the enrichment analysis, we simulated *N* = 100 input libraries for each ChIP-seq sample library. For each simulated input library, the number of read mappings contributing to a TE family/subfamily was computed as described above. An empirical *P-*value was computed for each TE family/subfamily as the number of simulated input libraries with the number of read mappings associated with the TE family/subfamily higher than or equal to the read mapping counting for the TE family/subfamily in the ChIP-seq sample library divided by *N*. TE families/subfamilies with *P*-value < 0.01 were considered enriched. A Fold-Change (FC) was calculated as the ratio between the read mapping counting for the TE family/subfamily in the ChIP-seq sample library divided by the average number of read mappings associated with the TE family/subfamily across all *N* simulated input libraries.

### Enrichment analysis using uniform background probability

Read mappings of the ChIP-seq sample library were randomly shuffled across the entire genome using BEDtools v2.27.1 function shuffle with default arguments [54]. Then, every position in the reference genome has the same probability for a read starting at this given position. The coordinates of the shuffled read mappings were then intersected with the repeat annotation using BEDMAP v2.4.37 [56] with options “--echo-map” to list all overlapping elements from repeat annotation file and “--echo-overlap-size” to output the lengths of overlaps in base pairs. Finally, we counted the number of mappings associated with each TE family/subfamily. The procedure was repeated *N* = 100 times. For each TE family/subfamily, an empirical *P*-value was calculated as the number of times when the number of read mappings associated with the TE family/subfamily was higher than or equal to the number of mappings for the TE family/subfamily in the ChIP-seq library divided by *N*.

### Software and computational specifications

T3E was developed for UNIX environments and it is freely available for download under GNU General Public License v3.0 at GitHub (https://github.com/michelleapaz/T3E). The program was written in Python (version 3.8.5). Auxiliary scripts were written in bash, Perl (version 5.30.0), and R (version 3.6.3). Instructions about usability, prerequisite software and libraries with the tested versions can be found at GitHub. The application was executed for samples running in parallel on a computer running Linux version 4.18.0 with in total 2 TB of RAM and with AMD EPYC 7542 32-Core Processor.

### Run-time and memory complexity analysis

We evaluated the run-time and memory complexity of our application by using the average-case scenario. We chose as average-case the ENCODE Chip-seq HepG2 sample and input control (Supplementary Table S1), both with read length of 75 bp. All the reads of the ChIP-seq sample library were considered. For the input control, we considered the entire library size of approximately 70 million reads (100%) and the library size down sampled to 80, 60, 40, 28.7% (20 million reads) and 20%. We measured the time of execution and peak memory consumption of T3E considering each input control library size subset using time and tracemalloc modules in Python 3.

## Supplementary Information

**Additional file 1.**

## Data Availability

No raw data were generated in the course of this study. T3E is available at https://github.com/michelleapaz/T3E.

## Abbreviations

ChIP-seq: Chromatin immunoprecipitation followed by high-throughput sequencing
CML: Chronic Myelogenous Leukemia
DCW: Dorsal Cerebral Wall
ENCODE: Encyclopedia of DNA Elements
ERVK: Endogenous retrovirus-K
FAM: Fossil Alu monomer
FC: Fold-Change
FOXP1: Transcription factor Forkhead box protein P1
GM12878: Lymphoblastoid cell
Hela: Human cervical adenocarcinoma cell
HepG2: Hepatocellular carcinoma cell
HERV: Human endogenous retrovirus
HUVEC: Human umbilical vein endothelial cell
IMR-90: Normal lung fibroblast cell
K562: Chronic myelogenous leukemia (CML) cell
LTR: Long terminal repeat
LINE: Long interspersed nuclear element
LINE-1: Long interspersed nuclear element-1
MER: Medium reiterated frequency repeats
PBDE: Peripheral blood-derived erythroblast
Pol II: RNA Polymerase II
Pol II S2: RNA Polymerase II phosphorylated on serine 2
Pol III: RNA Polymerase III
SINE: Short interspersed nuclear element
SVA: SINE-VNTR-Alu
TE: Transposable element
TFBS: Transcription factor binding sites
T3E: Transposable Element Enrichment Estimator
UCON: Ultra-conserved element
