# Supplementary material for: T3E: a tool for characterising the epigenetic profile of transposable elements using ChIP-seq data
Source: Mob DNA. 2022 Nov 30;13:29. doi: 10.1186/s13100-022-00285-z (PMC9710123; doi:10.1186/s13100-022-00285-z)
Supplement: Supplementary file 1 — Additional file 1. [file 13100_2022_285_MOESM1_ESM.pdf]

T3E: a tool for characterising the epigenetic profile of transposable elements using ChIP-seq data

Michelle Almeida da Paz and Leila Taher

Institute of Biomedical Informatics, TU Graz, Austria

SUPPLEMENTARY MATERIALS

Dataset	Sample	Target	File accession	Laboratory/ Reference	Read length (bp)	Library size	Input control
1	B-cell	NA	ENCFF000AMM	Bradley Bernstein (Broad)	35	43569325	-
			ENCFF001HAY	John Stamatoyannopoulos (UW)		26979122	
		H3K4me3	ENCFF001EUE		36	20123133	ENCFF001HAY
	HepG2	NA	ENCFF272WBE	Richard Myers (HAIB)	50	25554473	-
			ENCFF380HNN		100	26535355	
			ENCFF302HDW		75	69681898	
		FOXP1	ENCFF734HBO		75	18701212	ENCFF302HDW
	IMR-90	NA	ENCFF679UAT	Bing Ren (UCSD)	36	47554621	-
			ENCFF421HCG		35	10800072	
		H3K79me1	ENCFF682GXA			14050435	ENCFF679UAT, ENCFF421HCG
2	14.5 DCW	NA	SRR15671382	[27,28]	35	14251048	-
		p300	SRR15671381		36	17878738	SRR15671382
3	GM12878	NA	GM12878_input	[20]	27	8625420	-
		POL II	GM12878_pol2		35	15253593	GM12878_input
		POL II S2	GM12878_ pol2s2		36	32757721	
		POL III	GM12878_pol3		26	6013730	
	Hela	NA	Hela_input1		33	6133195	-
		POL II	Hela_pol2			4208492	Hela_input1
		NA	Hela_input2		35	24747965	-
		POL II S2	Hela_pol2s2			20297183	Hela_input2
		POL III	Hela_pol3			12763612	
	HUVEC	NA	Huvec_input		35	49566996	-
		POL II	Huvec_pol2		36	33228426	Huvec_input
	K562	NA	K562_input		35	17964555	-
		POL II	K562_pol2			26961880	K562_input
		H3K9me1	K562_ H3k9me1			24171879	
		H3K27ac	K562_ H3k27ac			14597481	
		H3K79me2	K562_ H3k79me2			37282045	
		H3K36me3	K562_ H3k36me3		34	9308987	
		H3K9me3	K562_ H3k9me3		36	37197946	
		H3K4me1	K562_ H3k4me1		38	12356771	
		H3K4me2	K562_ H3k4me2			8856317	
		H3K27me3	K562_ H3k27me3		41	18342667	
		H3K4me3	K562_ H3k4me3			19173297	
		H3K9ac	K562_ H3k9ac		47	13785094	
	PBDE	NA	Pbde_input		36	55388830	-
		POL II	Pbde_pol2			26082143	Pbde_input

Supplementary Table S1. Description of ChIP-seq datasets. Three datasets containing ChIP-seq experiments are detailed. Sample column refers to the cell type (cell line or primary cell). Input controls present no antibody targets (NA). Active chromatin histone marks (H3K27ac, H3K4me2, H3K9ac, H3K4me3, H3K79me1, H3K79me2, H3K4me1 and H3K36me3), repressive chromatin histone marks (H3K9me1, H3K9me3 and H3K27me3), transcription factor FOXP1, transcriptional co-activator p300, RNA Polymerases II, II S2 and III are targets used in ChIP-seq experiments. Entire names for file accessions are better described in Supplementary Table S2 for Dataset 3. Laboratory/Reference column refers to the research group that produced the ChIP-seq experiments or reference paper where the results were published. Read length in base pairs (bp) is calculated as the average of 10,000 reads of the library. Library sizes refer to the number of reads in the ChIP-seq library. In the input control column, hyphen represents input controls and ChIP-seq samples have their corresponding input control

file accession listed. The sample ENCFF682GXA has two corresponding input controls (ENCFF679UAT – “good control” and ENCFF421HCG – “bad control”).

Sample	File accession	Name accession
GM12878	GM12878_input	wgEncodeSydhTfbsGm12878IggmusRawDataRep1<br>wgEncodeSydhTfbsGm12878IggmusRawDataRep2
	GM12878_pol2	wgEncodeOpenChromChipGm12878Pol2RawDataRep2
	GM12878_ pol2s2	wgEncodeSydhTfbsGm12878Pol2s2IggmusRawDataRep1<br>wgEncodeSydhTfbsGm12878Pol2s2IggmusRawDataRep2
	GM12878_pol3	wgEncodeSydhTfbsGm12878Pol3RawDataRep1<br>wgEncodeSydhTfbsGm12878Pol3RawDataRep2
Hela	Hela_input1	wgEncodeOpenChromChipHelas3InputRawData
	Hela_pol2	wgEncodeOpenChromChipHelas3Pol2RawDataRep1
	Hela_input2	SRR036650<br>SRR036651<br>SRR036652<br>SRR036653<br>SRR036654
	Hela_pol2s2	wgEncodeSydhTfbsHelas3Pol2s2IggrabRawDataRep1
	Hela_pol3	SRR036655<br>SRR036657<br>SRR036658<br>SRR036659
HUVEC	Huvec_input	wgEncodeSydhTfbsHuvecInputUcdRawData
	Huvec_pol2	wgEncodeHaibTfbsHuvecPol2Pcr1xRawDataRep2
K562	K562_input	wgEncodeBroadHistoneK562ControlStdRawDataRep1
	K562_pol2	wgEncodeOpenChromChipK562Pol2RawDataRep1<br>wgEncodeOpenChromChipK562Pol2RawDataRep2
	K562_ H3k9me1	wgEncodeBroadHistoneK562H3k9me1StdRawDataRep1
	K562_ H3k27ac	wgEncodeBroadHistoneK562H3k27acStdRawDataRep1<br>wgEncodeBroadHistoneK562H3k27acStdRawDataRep2
	K562_ H3k79me2	wgEncodeBroadHistoneK562H3k79me2StdRawDataRep1<br>wgEncodeBroadHistoneK562H3k79me2StdRawDataRep2
	K562_ H3k36me3	wgEncodeBroadHistoneK562H3k36me3StdRawDataRep1<br>wgEncodeBroadHistoneK562H3k36me3StdRawDataRep2
	K562_ H3k9me3	wgEncodeBroadHistoneK562H3k9me3StdRawDataRep2
	K562_ H3k4me1	wgEncodeBroadHistoneK562H3k4me1StdRawDataRep1<br>wgEncodeBroadHistoneK562H3k4me1StdRawDataRep2
	K562_ H3k4me2	wgEncodeBroadHistoneK562H3k4me2StdRawDataRep1<br>wgEncodeBroadHistoneK562H3k4me2StdRawDataRep2
	K562_ H3k27me3	wgEncodeBroadHistoneK562H3k27me3StdRawDataRep1<br>wgEncodeBroadHistoneK562H3k27me3StdRawDataRep2
	K562_ H3k4me3	wgEncodeBroadHistoneK562H3k4me3StdRawDataRep1<br>wgEncodeBroadHistoneK562H3k4me3StdRawDataRep2
	K562_ H3k9ac	wgEncodeBroadHistoneK562H3k9acStdRawDataRep1
PBDE	Pbde_input	wgEncodeSydhTfbsPbdeInputUcdRawData
	Pbde_pol2	wgEncodeSydhTfbsPbdePol2UcdRawDataRep2

Supplementary Table S2. File accession description of Dataset 3. Entire names of ChIP-seq experiments are listed for each file accession. Files from the same file accession code are pooled together using SAMtools merge. Sample column refers to the different cell lines.

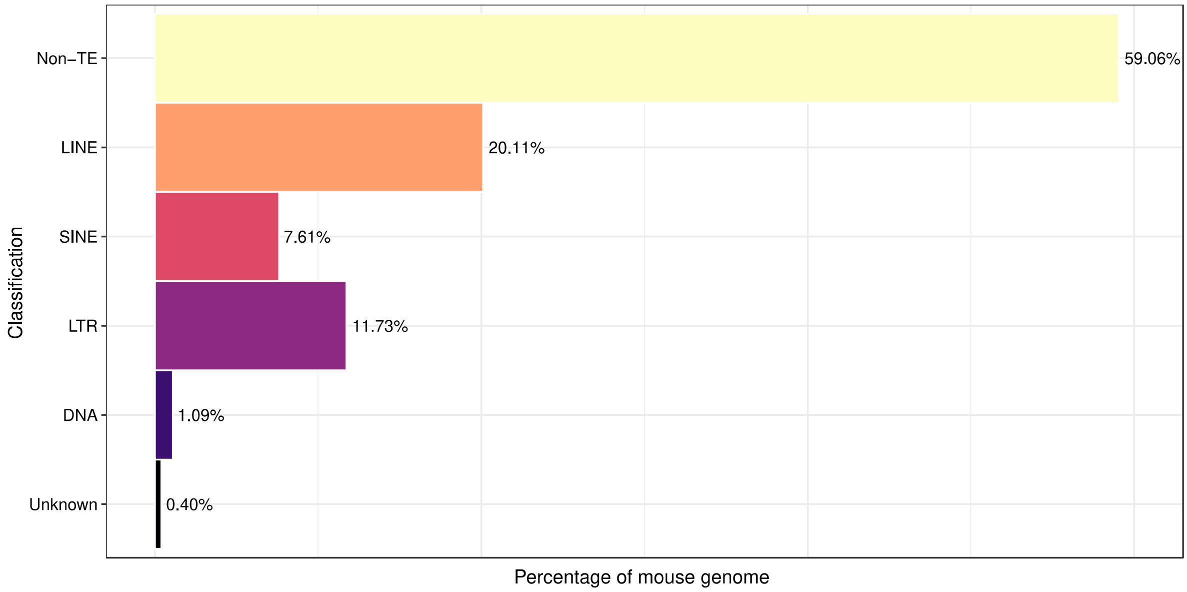

Supplementary Figure S1. Nearly half of the mouse genome is comprised by TEs. Percentage covered by TE classes and non TEs in the mouse genome. Colours represent different classifications of TEs (DNAs, LINEs, LTRs, SINEs) followed by their percentage in the mouse genome. TEs with unknown classification are registered as “Unknown”. The portion of the mouse genome not covered by TEs is indicated by “non-TE”.

A

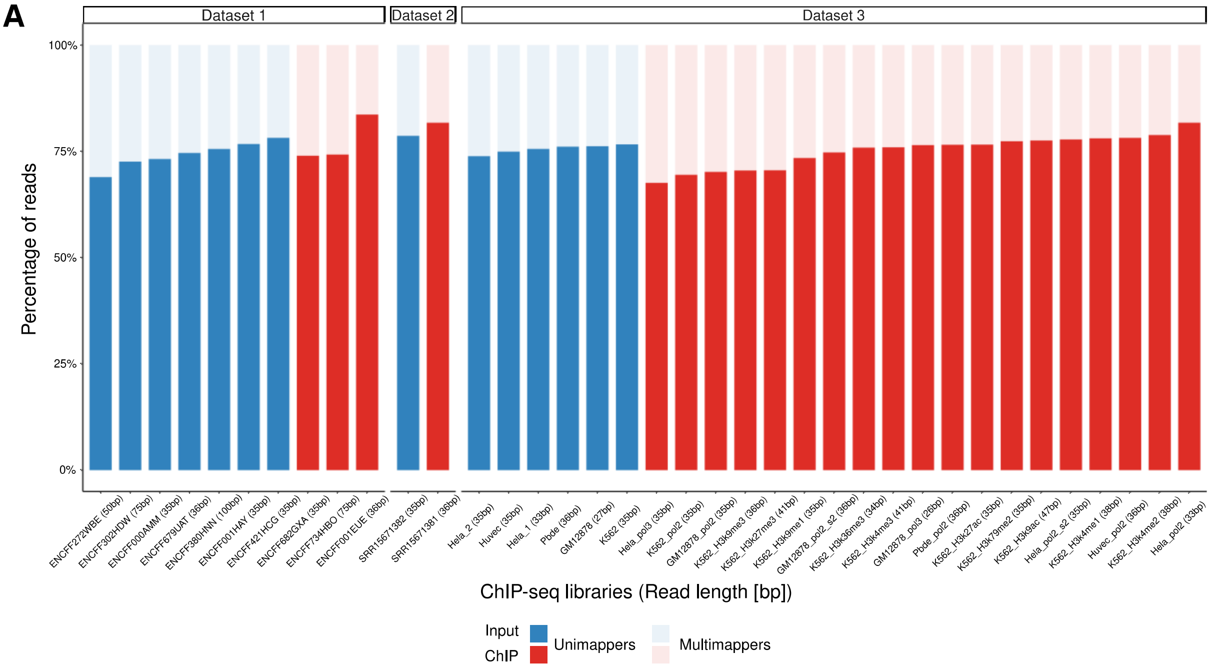

B

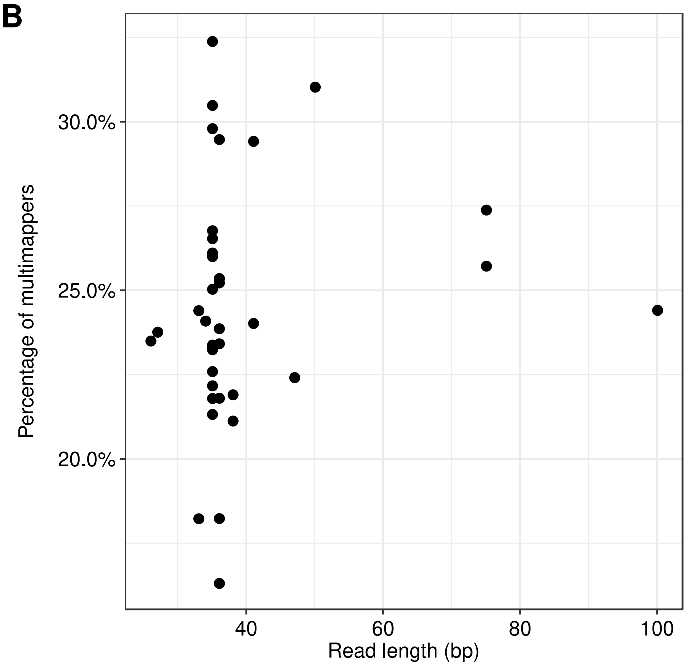

Supplementary Figure S2. Mapping statistics for all ChIP-seq experiments and their corresponding read lengths. (A) Fraction of uni- and multimappers for 37 ChIP-seq libraries (red) and their input controls (blue) (Datasets 1-3; Methods). Only small differences were found when comparing ChIP-seq input control experiments generated by the same laboratory (Richard Myers - HAIB) for the same cell type (HepG2) with 50 bp (ENCFF272WBE), 75 bp (ENCFF302HDW) and 100 bp (ENCFF380HNN), yielding 68.94%, 72.59% and 75.56% unimappers, respectively. (B) Read length alone does not explain

the differences observed in the percentage of yielded multimappers when comparing different ChIP-seq libraries within the range analysed here (26bp-100bp).

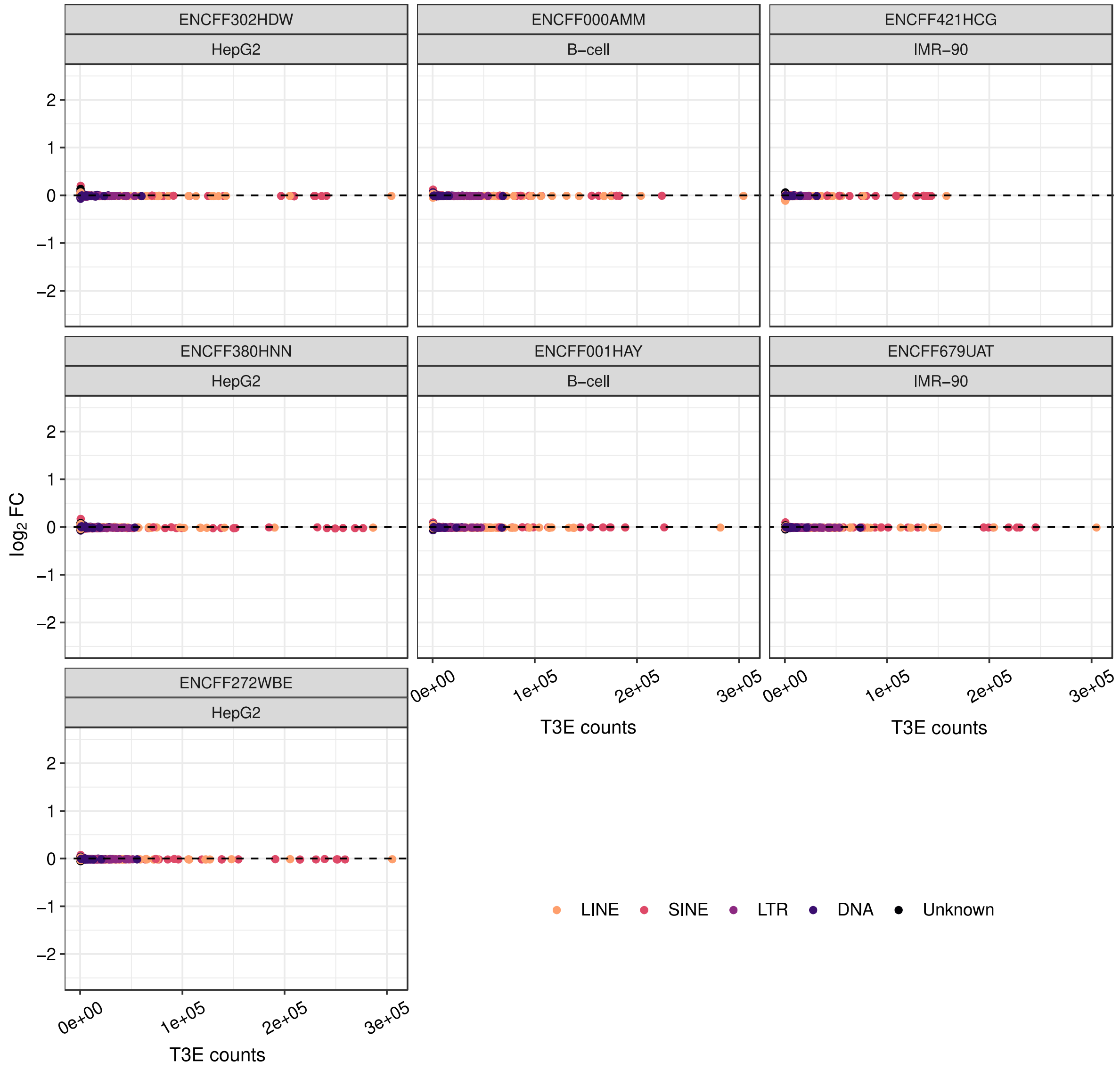

Supplementary Figure S3. The expected number of read mappings estimated by T3E reflects the input control. TE families/subfamilies are represented as dots and coloured according to their classification (i.e. DNA, LINE, LTR and SINE classes). TEs with unknown classification are registered as “Unknown”. Fold-Changes (FC) are calculated as the fraction among the average of read mapping counts from N = 100 T3E simulations and the read mapping counts of the input control DNA. Log_2FC is displayed on y-axis, while x-axis designates the average read mapping counts of T3E. Dashed line shows the ratio 1:1 of read mapping counts for T3E and input control. Seven ChIP-seq input control experiments from Dataset 1 are presented.

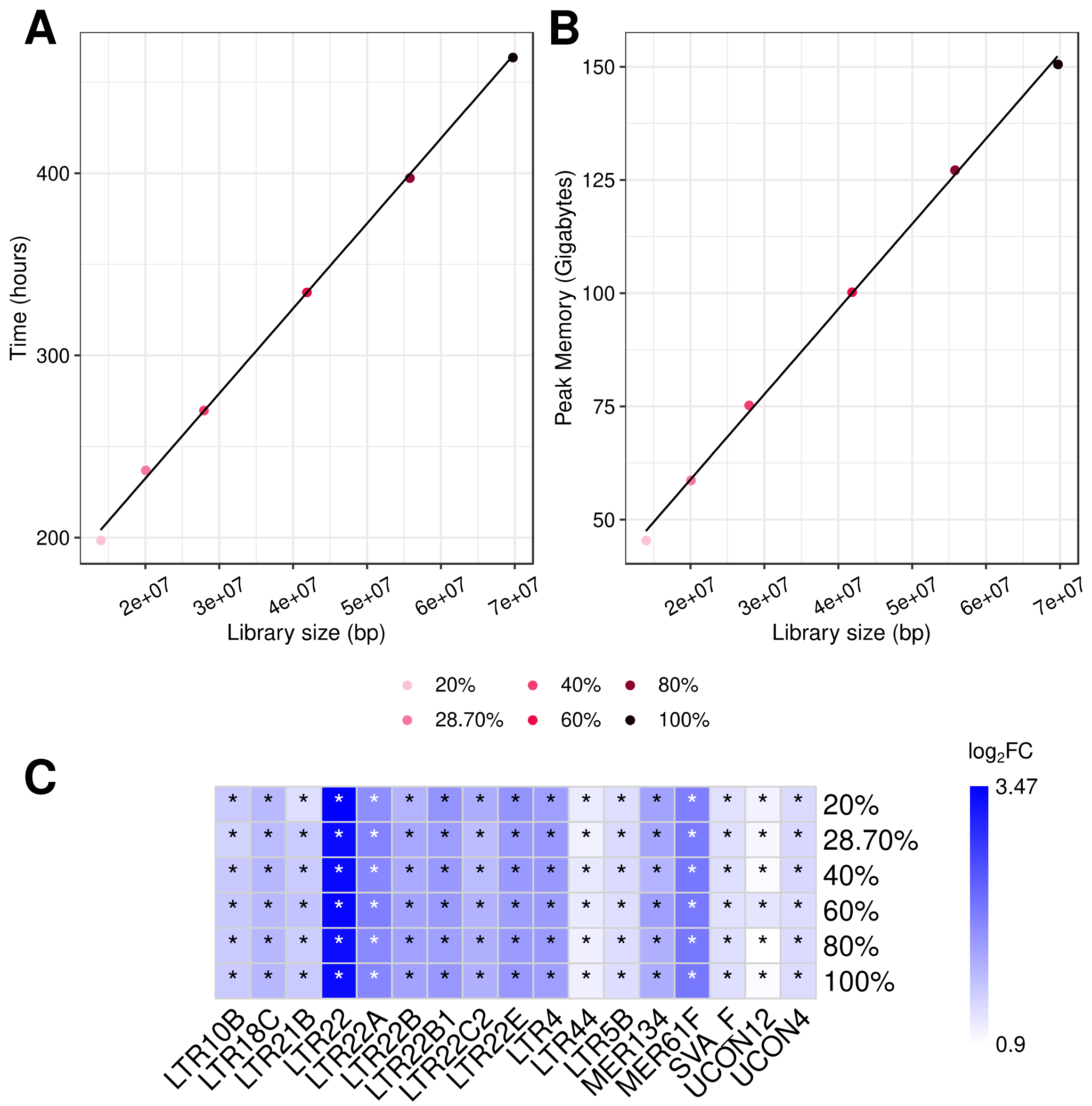

Supplementary Figure S4. Down-sampling input control still produces good results with T3E. Input library size of approximately 70 million reads (100%) and down-sampled library sizes (with their respective percentages to the actual input library size) are represented by different colours. The results of input library size of 20 million reads (28.70%) are described in Results section. T3E algorithm shows approximately linear run-time **(A)** and memory complexities **(B)** with respect to the input library size. Library size is represented in base pairs (bp). **(C)** Only enriched TE families/subfamilies (FC > 2 and P-value ≤ 0.05) of the HepG2 ChIP-seq sample are represented in the heatmap. Fold-changes are calculated as read mapping counts of the ChIP-seq sample divided by the average of read mapping counts of *N* = 100 simulated input libraries. P-values ≤ 0.05 are indicated by asterisks. White asterisks indicate FC > 4. Blue colour intensity varies according to the Log_2FC. The UCON12 subfamily is slightly affected by down-sampling since it contains only 56 individual copies in the human genome.

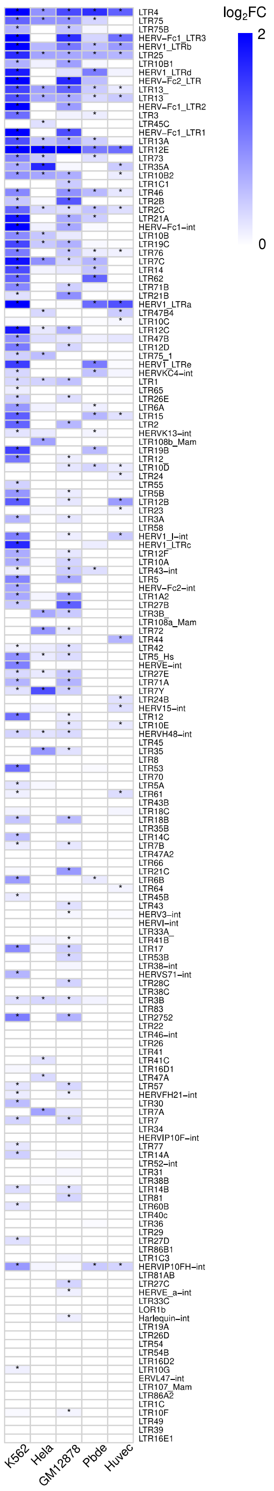

Supplementary Figure S5. RNA Polymerase II enrichment to LTR retrotransposons in different cell lines. Cancer (K562 and Hela), transformed (GM12878) and normal (PBDE and HUVEC) cell lines are displaced in columns in the heatmap. Only TE families/subfamilies described by repEnrich [20] are represented in the heatmap. P-values ≤ 0.05 are indicated by asterisks. Fold-changes are calculated as read mapping counts of the ChIP-seq sample divided by the average of read mapping counts of *N* = 100

simulated input libraries. Blue colour intensity varies according to the Log_2FC with a maximum of 2. White-coloured cells represent no signal.

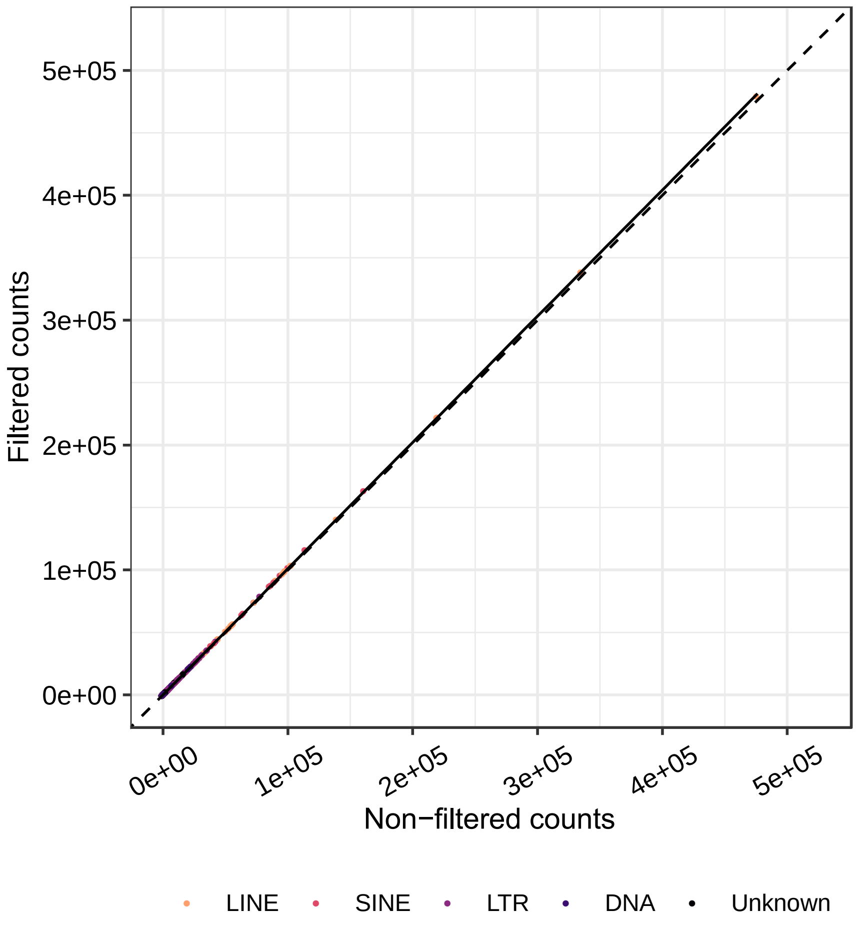

Supplementary Figure S6. Removing artifact regions from Dataset 2 does not impact on read mapping counts. Read mapping counts for each TE families/subfamilies calculated as the average of read mapping counts of *N* = 10 simulated input libraries using T3E. A linear one-to-one relationship among the non-filtered (x-axis) and filtered (y-axis) is observed suggesting that rarely filtered out regions comprehend a portion of individual copies of TE families/subfamilies. To illustrate, the filter approach only filtered out 0.001% of the entire region covered by any individual copy of the L1Md_F2 element, considered the TE with the highest number of read mapping counts and covering the highest number of nucleotides in the mouse genome. TE families/subfamilies are represented as dots and coloured according to their classification (i.e. DNA, LINE, LTR and SINE classes). TEs with unknown classification are registered as “Unknown”. Dashed line shows the diagonal and solid line indicates the fitted linear model.

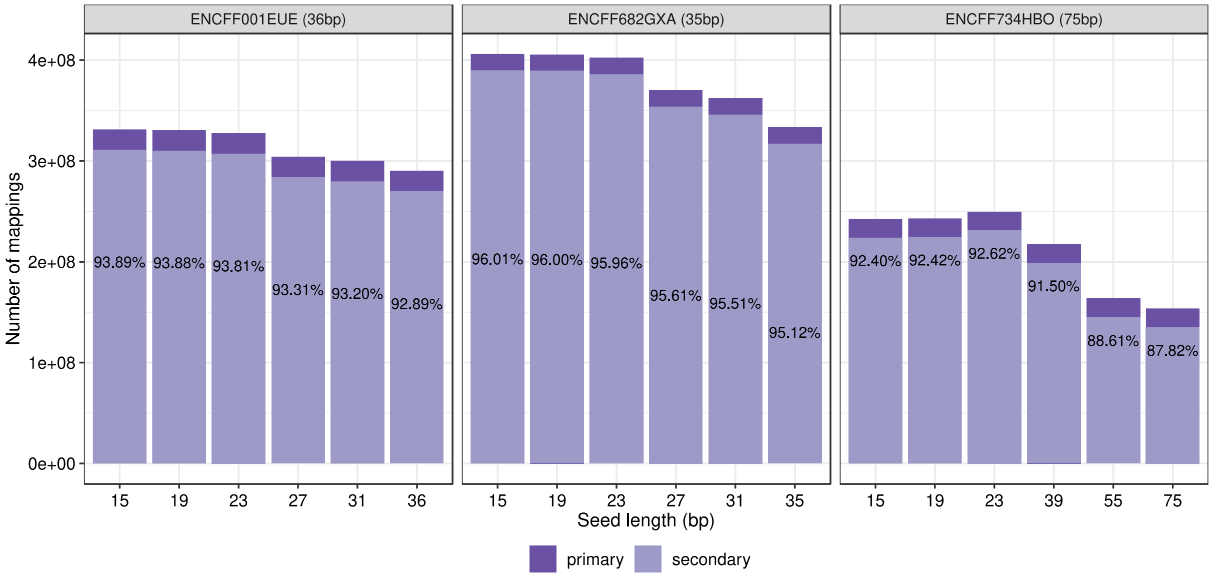

Supplementary Figure S7. Number of primary and secondary read mappings depending on the seed length parameter of bwa mem ("-k"). Mapping statistics obtained using the option "flagstat" of SAMtools. Six values were used for the parameter of bwa mem defining the minimum seed length ("-k"), starting from 15 and up to the actual read length of the ChIP-seq library. The default is "-k 19". Increasing the minimum seed length from the default value "-k 19" reduces the number of secondary mappings and, overall, the sensitivity. Secondary mappings are indicated as percentages.

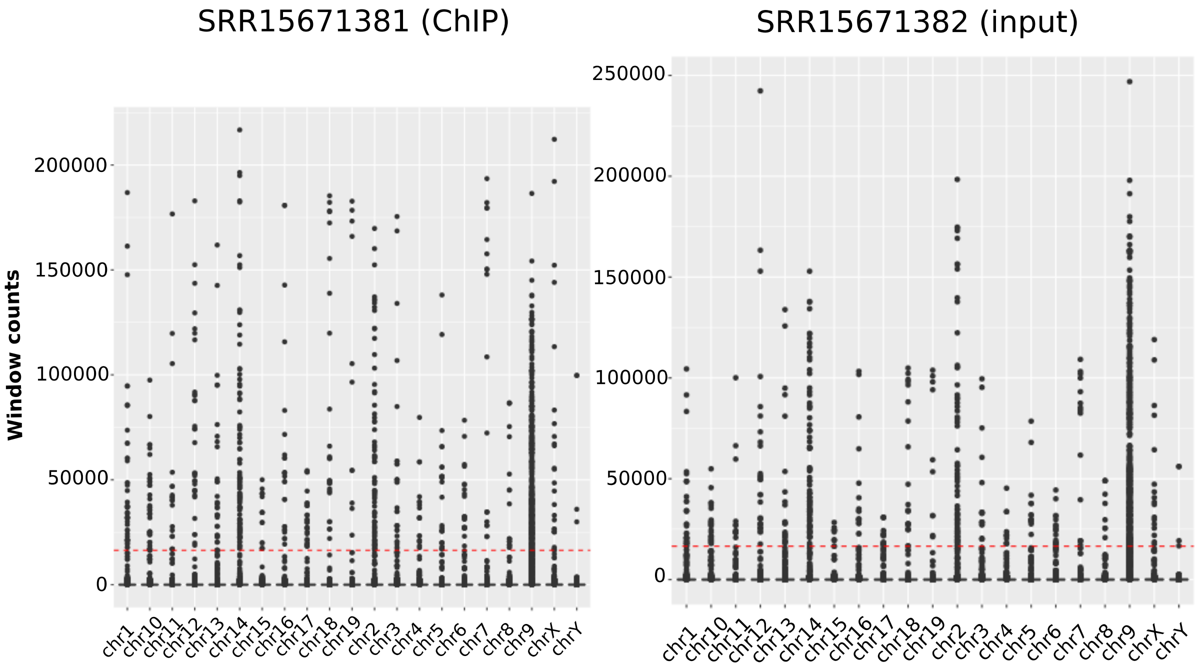

Supplementary Figure S8. Window read mapping counts for ChIP-seq sample and input control of Dataset 2. Sliding windows of size 100 bp were created scanning each chromosome of the mouse genome considering 50 bp of step. Dashed red line represent the threshold of 99.997th percentile considering both chromosome and genome.
